# Perturbing the Normal Level of SIDT1 Suppresses the Naked ASO Effect

**DOI:** 10.1155/2021/2458470

**Published:** 2021-11-16

**Authors:** Masayuki Takahashi, Mineaki Seki, Masayuki Nashimoto, Tomohiro Kabuta

**Affiliations:** ^1^Research Institute for Healthy Living, Niigata University of Pharmacy and Applied Life Sciences, Niigata, Niigata, Japan; ^2^Department of Degenerative Neurological Diseases, National Institute of Neuroscience, National Center of Neurology and Psychiatry, Kodaira, Tokyo, Japan

## Abstract

Although antisense oligonucleotide (ASO) therapeutics can be taken up by living cells without carrier molecules, a large part of incorporated ASOs are trapped in the endosomes and do not exert therapeutic effects. To improve their therapeutic effects, it would be important to elucidate the mechanism of cellular uptake and intracellular trafficking of ASOs. In this study, we investigated how SIDT1 affects cellular uptake and intracellular trafficking of ASOs. Fluorescence microscopic analysis suggested that most of naked ASOs are trafficked to the lysosomes via the endosomes. The data obtained from flow cytometry and fluorescence microscopy together showed that although the SIDT1 level barely affects the total cellular uptake of ASOs, it appears to affect the intracellular trafficking of ASOs. We also showed that SIDT1 exists mainly in the endoplasmic reticulum and that perturbing the normal level of SIDT1 suppresses the antisense effect of the naked ASO targeting miR-16.

## 1. Introduction

Antisense oligonucleotide (ASO) therapeutics can be taken up by living cells without carrier molecules such as cationic lipids, and they can act as gene silencing triggers or exon skipping inducers [1]. However, a large part of incorporated ASOs do not exert the therapeutic effects in cells, since they are trapped in the endosomes after being incorporated into cells and are eventually degraded in the lysosomes [1]. Although such a “nonproductive uptake” phenomenon is common, part of ASO molecules can escape from the endosomes and exert the therapeutic effect [1, 2].

In order to improve the therapeutic effects of ASOs, it would be important to elucidate the mechanism of cellular uptake and intracellular trafficking of ASOs. ASOs are thought to be taken up by cells via endocytosis [1], in which adaptor-related protein complex 2 subunit mu 1 (AP2M1) appears to be involved [3]. Coat protein complex II (COPII), which transports proteins from the rough endoplasmic reticulum (ER) to the Golgi apparatus, has been reported to be involved in the endosomal escape of ASOs [4].

Shih et al. have shown that the *C. elegans* systemic RNA interference-deficient 1 (SID-1) protein in the plasma membranes functions as a bidirectional transporter for double-stranded RNA (dsRNA) but not for single-stranded RNA (ssRNA) [5]. A mammalian ortholog of SID-1, SID-1 transmembrane family member 2 (SIDT2), is expressed in many types of cells and mediates the uptake of ssRNA into the lysosomes [6–10]. We have also reported that SIDT2 appeared to be involved in cellular uptake of 2′-*O*-methylated ASOs [11]. In this study, we investigated how SIDT1, a homolog of SIDT2, affects cellular uptake and intracellular trafficking of 2′-*O*-methylated ASOs.

## 2. Materials and Methods

### 2.1. Cell Culture

Mouse embryonic fibroblasts (MEFs) were cultured in Dulbecco's modified Eagle's medium (Thermo Fisher Scientific, C11995500BT) supplemented with 10% fetal bovine serum (Sigma-Aldrich, 172012) at 37°C in a 5% CO_2_ humidified incubator [10]. Cell passage was performed every time the cells reached 80 to 100% confluency.

### 2.2. Preparation of Oligonucleotides and Plasmids

The fully 2′-*O*-methylated, 5′-Alexa568-labeled, 3′-phosphorylated ASO A-15 (5′-AAAAAAAAAAAAAAA-3′) and the fully 2′-*O*-methylated, 5′- and 3′-phosphorylated miR-16-targeting ASO antimir-16 (5′-CGCCAAUAUUUACGUGCUGCUA-3′) were chemically synthesized and subsequently purified by high-performance liquid chromatography as a custom service by Nippon Bioservice (Saitama, Japan).

The following primers used for quantitative RT-PCR (qPCR) were obtained from Fasmac or Hokkaido System Science (Hokkaido, Japan): *β*-actin forward primer, 5′-CGTGCGTGACATCAAAGAGAA-3′; *β*-actin reverse primer, 5′-CAATAGTGATGACCTGGCCGT-3′; miR-16 reverse transcription primer, 5′-GTCGTATCCAGTGCAGGGTCCGAGGTATTCGCACTGGATACGACCGCCAA-3′; miR-16 forward primer, 5′-CGCGCTAGCAGCACGTAAAT-3′; miR-16 reverse primer, 5′-GTGCAGGGTCCGAGGTATTCG-3′.

The siRNAs siSIDT1#1 (the sense sequence: 5′-GGCUUACCCGUGUUCAGUU-3′), siSIDT1#2 (the sense sequence: 5′-CUUAGGGGACCGAACUCCU-3′), and siControl (the sense sequence: 5′-GCCACAACGUCUAUAUCAU-3′) were chemically synthesized by Nippon Bioservice (Saitama, Japan).

The mouse SIDT1 (NP_932151.2) expression plasmids pCI-neo-SIDT1 and pEGFP-N1-SIDT1 were prepared as described previously [10].

### 2.3. Transfection

Transfection experiments were conducted using Lipofectamine 3000 reagent (Thermo Fisher Scientific, L3000015) in accordance with manufacturer's protocol (forward transfection). Cells were cultured for 72 and 48 hours after transfection with siRNA and plasmid, respectively, and then used for various assays.

### 2.4. Quantitative RT-PCR

Total RNA was extracted from MEFs using TRIzol (Thermo Fisher Scientific, 15596018) or RNAiso Plus (Takara, 9108), and cDNA was synthesized using PrimeScript™ RT Reagent Kit with gDNA Eraser (Perfect Real Time) (Takara, RR047A), in accordance with manufacturer's protocols. *β*-Actin mRNA and miR-16 levels were quantified using a CFX96TM Real-Time System (Bio-Rad) or Thermal Cycler Dice Real Time System (Takara) with SYBR Premix Ex Taq™ II (Tli RNaseH Plus) (Takara, RR0820A) [11].

### 2.5. Confocal Fluorescence Microscopy

MEFs prepared in 35 mm glass-bottomed dishes (IWAKI, 3910035 or ibidi, 81158) were cultured as above. After treating with various reagents for the indicated time, the cells were washed with PBS and analyzed using a FLUOVIEW FV10i or FV1000 confocal microscope (Olympus). The fluorescence intensity was quantified with ImageJ (NIH). Each field of view was comprised of ~100−150 cells, and at least 3 fields of view per condition were quantified. ER-Tracker™ Red (Thermo Fisher Scientific, E34250) was used to stain the ER, and LysoTracker™ Green DND-26 (Thermo Fisher Scientific, L7526) and LysoTracker™ Red DND-99 (Thermo Fisher Scientific, L7528) were to stain the lysosomes.

### 2.6. Flow Cytometry

MEFs were cultured for 6 or 24 hours after adding 500 nM of naked A-15 to the medium. Then, the cells were washed with PBS and analyzed with a flow cytometer, SH800Z (SONY).

### 2.7. Western Blotting

Sodium dodecyl sulfate polyacrylamide gel electrophoresis and Western blotting were performed according to standard procedures [11]. Antibodies used were the polyclonal antibody against SIDT1 (Sigma Aldrich, SAB2501860) and the monoclonal antibody against *β*-actin (Sigma Aldrich, A4700).

### 2.8. Statistical Analysis

For comparison of two groups, the statistical significance of difference was evaluated by Student's *t*-test.

## 3. Results

### 3.1. Naked ASOs Are Mainly Localized to the Lysosomes

**Figure 1:**
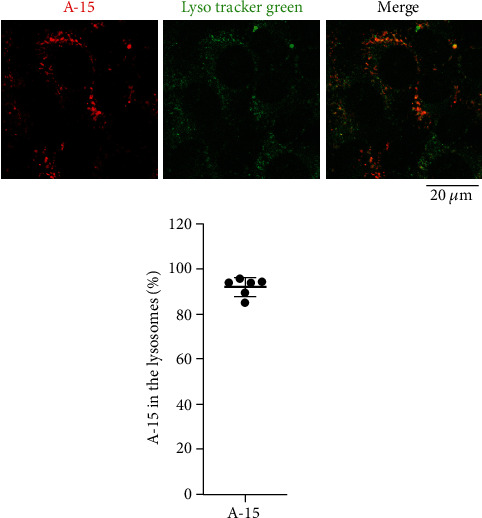
Subcellular location of incorporated naked A-15. After 24-hour culture in the presence of naked Alexa568-labeled A-15 (500 nM), MEFs were rinsed with PBS, and fluorescence images from Alexa568 and LysoTracker Green were visualized using a confocal microscope. Colocalization rates were quantified with ImageJ. Error bars indicate s.d. (*n* = 6).

First of all, we examined subcellular localization of naked ASOs incorporated into MEFs by confocal fluorescence microscopy. MEFs were cultured in the presence of a naked ASO, A-15. We observed that A-15 was taken up by MEFs, and on average, ~95% of A-15 molecules incorporated appeared to be localized to the lysosomes (Figure 1). This result suggests that most of the naked ASOs are trafficked to the lysosomes via the endosomes.

### 3.2. The Effect of SIDT1 on Total Cellular Uptake of the Naked ASO

**Figure 2:**
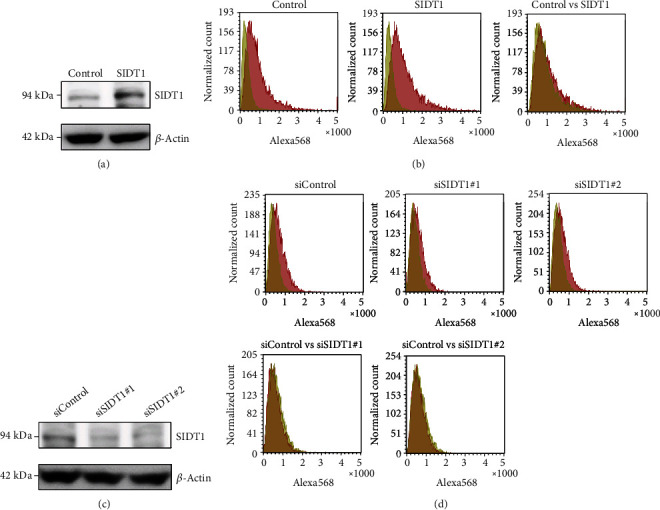
The effect of SIDT1 on total cellular uptake of naked A-15. (a) MEFs were transfected with the empty plasmid (control) or pCI-neo-SIDT1 for 48 hours, and the SIDT1 expression level was analyzed by Western blotting. (b) MEFs were transfected with the empty plasmid (control) or pCI-neo-SIDT1. After the 48-hour culture, the cells were rinsed with PBS and further cultured in the absence or presence of 500 nM of naked A-15 for 24 hours. After washing the cells with PBS, they were analyzed by flow cytometry. In the left and middle panels, yellow and red histograms are from MEFs cultured in the absence and presence, respectively, of A-15. In the right panel, the red histograms in the left and middle panels are overlaid and shown in yellow and red, respectively. (c) MEFs were transfected with the control siRNA (siControl) or the siRNA against the SIDT1 mRNA (siSIDT1#1 or siSIDT1#2) for 72 hours, and the SIDT1 expression level was analyzed by Western blotting. (d) MEFs were transfected with siControl, siSIDT1#1, or siSIDT1#2. After the 72-hour culture, the cells were rinsed with PBS and further cultured in the absence or presence of 500 nM of naked A-15 for 6 hours. After washing the cells with PBS, they were analyzed by flow cytometry. In the upper panels, yellow and red histograms are from MEFs cultured in the absence and presence, respectively, of A-15. In the lower left panel, the red histograms in the upper left and middle panels are overlaid and shown in yellow and red, respectively. In the lower right panel, the red histograms in the upper left and right panels are overlaid and shown in yellow and red, respectively.

We examined whether overexpression of SIDT1 enhances total uptake of the naked A-15 into cells. MEFs, which were transfected with the SIDT1-expressing plasmid, were incubated with or without A-15 and analyzed by flow cytometry (Figures 2(a) and 2(b)). Although Alexa568 signals were not completely separated from autofluorescence, the distribution in the SIDT1-overexpressing cells was slightly shifted to the right compared with that in the control cells (Figure 2(b)).

We tested how the total uptake of the naked A-15 is affected in SIDT1-knockdown cells. We transfected MEFs with siRNAs against the SIDT1 mRNA and confirmed that the siRNAs reduce the SIDT1 protein level (Figure 2(c)). We cultured MEFs in the presence or absence of the naked A-15 for 6 hours and analyzed them by flow cytometry. The distributions in the cytometric histograms for the SIDT1 knockdown cells were slightly shifted to the left compared with that for the control cells (Figure 2(d)). These results indicated that the SIDT1 level barely affects the total cellular uptake of the naked A-15.

### 3.3. Microscopic Analysis for the Cellular Uptake of the Naked ASO

**Figure 3:**
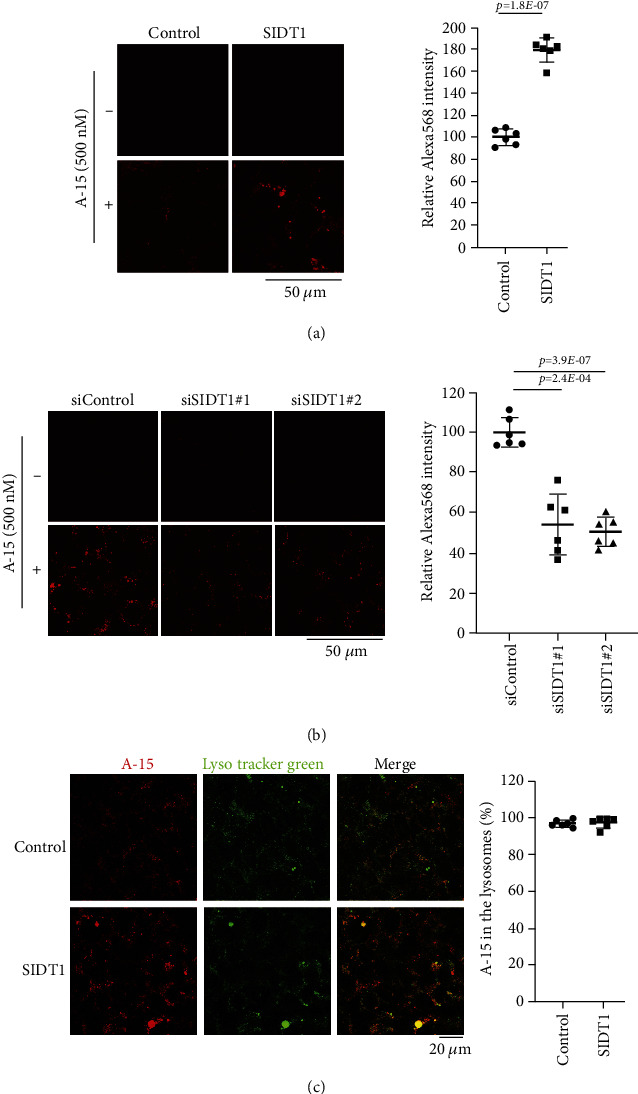
Microscopic analysis for the cellular uptake of naked Alexa568-labeled A-15. (a) MEFs were transfected with the empty plasmid (control) or pCI-neo-SIDT1. After the 48-hour culture, the cells were rinsed with PBS and further cultured in the absence (–) or presence (+) of 500 nM of naked A-15 for 24 hours. After washing the cells with PBS, they were analyzed by confocal microscopy. The Alexa568 fluorescence intensity in the SIDT1-overexpressing cells relative to that in the control cells is shown. Error bars indicate s.d. (*n* = 6). (b) MEFs were transfected with siControl, siSIDT1#1, or siSIDT1#2. After the 72-hour culture, the cells were rinsed with PBS and further cultured in the absence (–) or presence (+) of 500 nM of naked A-15 for 6 hours. After washing the cells with PBS, they were analyzed by confocal microscopy. The Alexa568 fluorescence intensity in the SIDT1 knockdown cells relative to that in the control cells is shown. Error bars indicate s.d. (*n* = 6). (c) MEFs were transfected with the empty plasmid (control) or pCI-neo-SIDT1. After the 48-hour culture, the cells were rinsed with PBS and further cultured in the presence of 500 nM of naked Alexa568-labeled A-15 for 24 hours. After washing the cells with PBS, fluorescence images from Alexa568 and LysoTracker Green were visualized using a confocal microscope. Colocalization rates were quantified with ImageJ. Error bars indicate s.d. (*n* = 6).

We next examined microscopically how the SIDT1 level affects cellular uptake of ASO. SIDT1-overexpressing or SIDT1 knockdown MEFs were incubated with the naked A-15 and analyzed by confocal fluorescence microscopy. The level of fluorescence intensity in the SIDT1-overexpressing cells was increased on average by ~80% compared with that in the control cells (Figure 3(a)). We also observed that the level of fluorescence intensity in the SIDT1 knockdown cells was decreased on average by ~50% compared with that in the control cells (Figure 3(b)).

We confirmed that on average, ~97% of the A-15 molecules were localized to the lysosomes in the SIDT1-overexpressing MEFs like in the control cells (Figures 1 and 3(c)). As discussed below, these results together with the cytometric data suggested that although the SIDT1 level barely affects the total cellular uptake of ASO, it appears to affect the intracellular trafficking of ASO.

### 3.4. SIDT1 Exists Mainly in the Endoplasmic Reticulum

**Figure 4:**
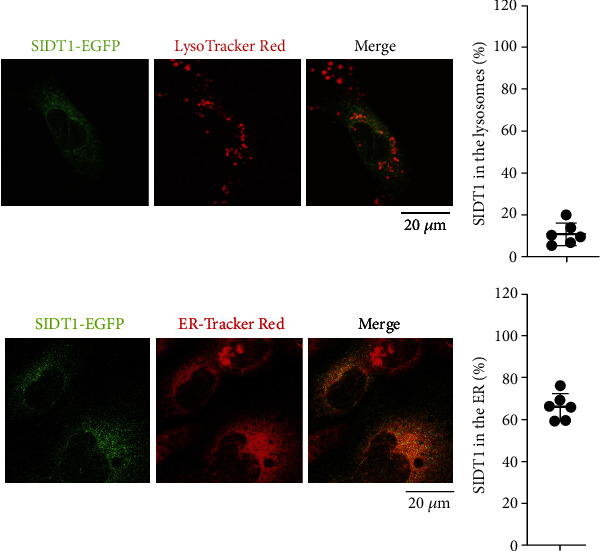
Localization analysis for SIDT1. MEFs were transfected with pEGFP-N1-SIDT1 and cultured for 24 hours. Then, MEFs were stained with LysoTracker Red or ER-Tracker Red, and fluorescence images were visualized using a confocal microscope. The colocalization rates were quantified with ImageJ. Error bars indicate s.d. (*n* = 6).

We performed intracellular localization analysis using the C-terminal EGFP-tagged SIDT1. We transfected MEFs with its expression plasmid, cultured them for 24 hours, and located the SIDT1 by costaining the lysosomes or the endoplasmic reticulum (ER) with a confocal fluorescence microscope. About 65% of the EGFP-tagged SIDT1 were localized to the ER, and ~10% existed in the lysosomes (Figure 4).

### 3.5. Perturbing the Normal Level of SIDT1 Dampens the Naked ASO Effect

**Figure 5:**
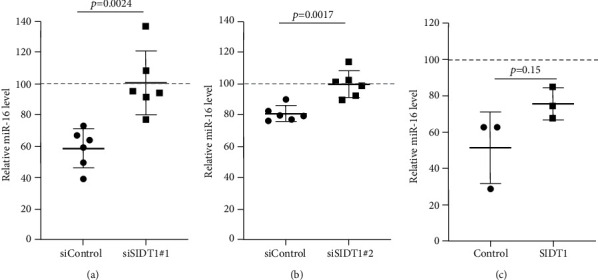
Perturbing the normal level of SIDT1 dampens the antimir-16 effect. (a) and (b) MEFs were transfected with siControl, siSIDT1#1, or siSIDT1#2 for 72 hours. Then, cells were rinsed with PBS and cultured with (+) or without (–) antimir-16 (20 nM) for 6 hours. miR-16 levels were analyzed by qPCR and normalized against *β*-actin mRNA levels. The miR-16 levels are expressed as percentages relative to those in MEFs untreated with antimir-16. Error bars indicate s.d. (*n* = 6). (c) MEFs were transfected with the empty plasmid or pCI-neo-SIDT1 for 48 hours. Cells were rinsed with PBS, and cultured with (+) or without (–) antimir-16 (20 nM) for 6 hours. The miR-16 levels were analyzed as above. Error bars indicate s.d. (*n* = 3).

Next, we examined how the SIDT1 level affects the effect of the ASO antimir-16 targeting miR-16. The SIDT1 knockdown MEFs were incubated with or without naked antimir-16, and the level of miR-16 was analyzed by qPCR. We observed that decreasing the SIDT1 level dampens the antisense effect of antimir-16 by ~25−67% (Figures 5(a) and 5(b)). The SIDT1-overexpressing cells were also examined for the antimir-16 effect, and its antisense effect was dampened again, albeit statistically insignificantly (Figure 5(c)). These results suggest that perturbing the normal level of SIDT1 suppresses the naked ASO effect.

### 3.6. The SIDT2 Level Hardly Affects the Total Cellular Uptake of ASOs

**Figure 6:**
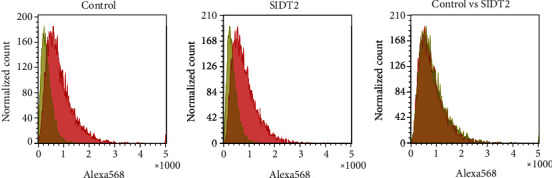
The SIDT2 level hardly affects the total cellular uptake of naked A-15. MEFs were transfected with the empty plasmid (control) or pCI-neo-SIDT2 for 48 hours. Then, the cells were rinsed with PBS and cultured with or without 500 nM of naked A-15. After the 24-hour culture, the cells were washed with PBS and analyzed with a flow cytometer. In the left and middle panels, yellow and red histograms are from MEFs cultured in the absence and presence, respectively, of A-15. In the right panel, the red histograms in the left and middle panels are overlaid and shown in yellow and red, respectively.

Previously, only from the microscopic analysis, we reported that SIDT2, a homolog of SIDT1, mediated the cellular uptake of A-15 [11]. Here, we cytometrically reexamined the effect of SIDT2 on the A-15 uptake. SIDT2-overexpressing MEFs were incubated with naked A-15 and analyzed by flow cytometry. The distributions in the cytometric histograms showed almost no difference between control and SIDT2-overexpressing cells (Figure 6). This result and our previous microscopic data together suggest that the SIDT2 level hardly affects the total cellular uptake of ASOs, whereas it appears to affect the intracellular trafficking of ASOs, in a similar fashion to SIDT1.

## 4. Discussion

### 4.1. Fluorescence Microscopy and Flow Cytometry Are Complementary

From the previous reports [1, 12, 13], we have learned that fluorescence microscopic analysis for the total cellular uptake of ASOs tends to underestimate the actual amount of incorporated ASOs compared with the flow cytometric analysis. This would be because the microscope has difficulty in detecting fluorescence signals from fluorophores dispersed throughout the cytosol and the nucleosol compared with those from fluorophores concentrated in the organelles. Cytometry and microscopy would be appropriate for analyzing the total cellular uptake of ASOs and its subcellular localization/trafficking, respectively. In this study, we performed both analyses for naked ASOs and concluded that the SIDT1 level hardly affects the total cellular uptake of A-15, but affects its subcellular distribution. The higher the SIDT1 level is, the more A-15 molecules would be concentrated in the lysosomes. This observation on SIDT1 urged us to reexamine SIDT2 for its role in ASO uptake, and the current data suggests that, contrary to the previous supposition, the SIDT2 level hardly affects the total cellular uptake of A-15, but appears to affect its subcellular distribution.

### 4.2. Suppression of the ASO Effect by Increasing the SIDT1 Level

**Figure 7:**
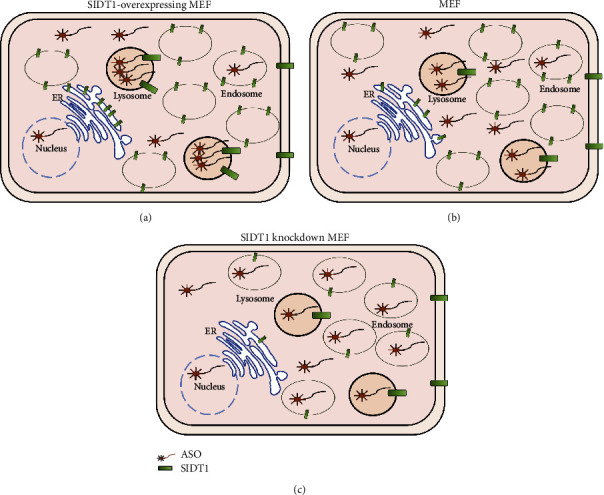
Aberrant levels of SIDT1 suppress the naked ASO effect. SIDT1-overexpressing (a), normal (b), and SIDT1 knockdown (c) MEFs are shown. The numbers of ASO, SIDT1, and SIDT2 molecules reflect relative amounts among in those cells, and only the ASO molecules in the cytosol and the nucleosol are supposed to exert the antisense effect.

The antisense effect of antimir-16 was suppressed by increasing the SIDT1 level. This may be attributed to an increased amount of lysosome-trapped antimir-16 (Figures 7(a) and 7(b)). The increase in the SIDT1 level in the ER and the lysosomes may stimulate the endosome maturation to the lysosomes and dampen the endosomal escape of ASO through the interaction of the ER with the endosomes/lysosomes somehow via SIDT1 [14, 15].

### 4.3. Suppression of the ASO Effect in the SIDT1 Knockdown Cells

The antimir-16 effect was dampened in the SIDT1 knockdown MEFs. This observation cannot be explained as above by simply considering the ASO amount in the lysosomes, since the level of lysosome-trapped ASO was decreased in the SIDT1 knockdown cells. It would be likely that a part of the antimir-16 molecules were trapped in another organelle, probably the endosomes, resulting in a decreased amount of free cytosolic antimir-16 that can target miR-16 (Figures 7(b) and 7(c)), although their existence in the endosomes was not examined. One of the SIDT1 splicing variants that exists in the endosomes has been shown to be able to export dsRNA to cytosol [16]. Thus, concomitant downregulation of this variant by the siRNAs may have decreased the level of endosomal-escaped antimir-16 and dampened the antisense effect.

### 4.4. Locations and Functions of SIDT1

Several findings by us and other groups on the location and the function of mouse and human SIDT1 are listed in Table 1. SIDT1 is reported to exist in the plasma membrane, the ER, the lysosomes, the endosomes, and the Golgi apparatus [12, 13, 16, 17]. There appears to be two forms (accession numbers NP_932151.2 and EDK98036.1) in mouse cells and only one form (accession numbers NP_060169.2) that corresponds to the mouse former form in human cells. Only the mouse latter form, which has an additional 8 amino acids in the middle of the first extramembrane loop, appears to exist in the endosomes [12]. With respect to the functions involved in nucleic acids, SIDT1 in the plasma membrane, the ER, and the endosomes appears to function for oligonucleotide uptake/transport [13, 17–19], endosome maturation, and endosomal escape [12], respectively. SIDT1 has been also reported to operate to take up and traffic cholesterol [16]. Apparent discrepancies among the reports would be in part due to the differences in cell types, nucleic acid types, SIDT1 forms, and/or experimental conditions.

### 4.5. Perspective

Although SIDT1 appears to play various cell physiological roles including cellular uptake and intracellular trafficking of oligonucleotides, its detailed functions remain to be elucidated. To elucidate the whole picture of the productive uptake of ASOs is much more challenging. New findings on its mechanism would help develop novel ASOs that can more efficiently exert the therapeutic effect.

## Figures and Tables

**Table 1:** Locations and functions of SIDT1.

Species	Claimed function	Location	Splicing variant	Reference
Mouse	Endosome maturation	ER, lysosome	NP_932151.2	This study
	Endosomal escape of poly (I : C)	Endosome, lysosome	EDK98036.1	12
	miRNA uptake	Plasma membrane	—	13
Human	siRNA uptake	Plasma membrane, ER, Golgi	NP_060169.2	17
	Intercellular transport of miRNA	Plasma membrane	NP_060169.2	18
	Cholesterol uptake and trafficking	Plasma membrane, ER	—	16
	miRNA uptake	Plasma membrane	—	19

## Data Availability

No data were used to support this study.

## Abbreviations

ASO: Antisense oligonucleotide
AP2M1: Adaptor-related protein complex 2 subunit mu 1
COP*ΙΙ*: Coat protein complex *ΙΙ*
ER: Endoplasmic reticulum
dsRNA: Double-stranded RNA
ssRNA: Single-stranded RNA
SID-1: Systemic RNA interference-deficient 1
SIDT2: SID-1 transmembrane family member 2
SIDT1: SID-1 transmembrane family member 1
MEF: Mouse embryonic fibroblast
qPCR: Quantitative RT-PCR
antimir-16: miR-16-targeting ASO.
